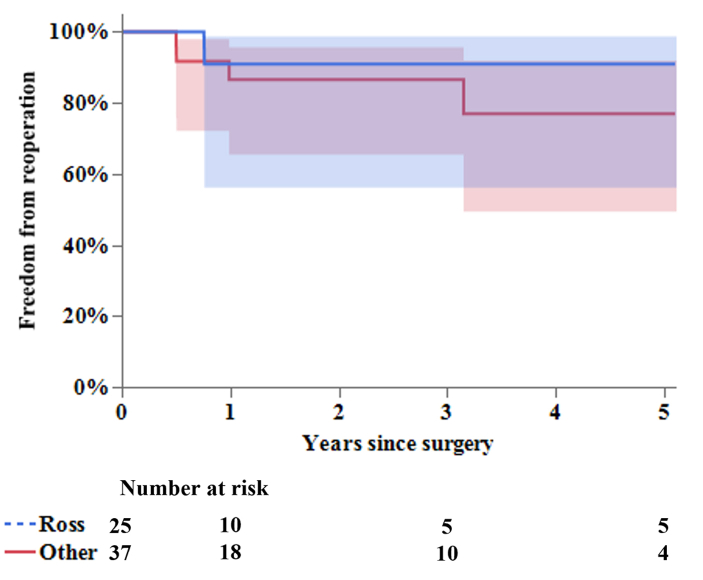# The Ross Procedure in Active Infective Endocarditis: A Comparison With Conventional Prostheses

**DOI:** 10.1016/j.atssr.2024.08.011

**Published:** 2024-09-01

**Authors:** Hiromu Kehara, Mohammed Kashem, Huaqing Zhao, Sebastian A. Iturra, Suyog A. Mokashi, Ravishankar Raman, Roh Yanagida, Kewal Krishan, Norihisa Shigemura, Yoshiya Toyoda

**Affiliations:** 1 Division of Cardiovascular Surgery, Lewis Katz School of Medicine at Temple University, Philadelphia, Pennsylvania; 2 Center for Biostatistics and Epidemiology, Department of Biomedical Education and Data Science, Lewis Katz School of Medicine at Temple University, Philadelphia, Pennsylvania

## Abstract

**Background:**

The Ross procedure can be an ideal option in infective endocarditis (IE) due to greater resistance to infection. However, limited literature has highlighted the comparison with conventional prostheses in this setting.

**Methods:**

Between February 2013 and September 2022, 25 patients (median age, 50 years) underwent a Ross procedure for IE (Ross group). The results were compared with those after other aortic valve procedures (aortic valve replacement and aortic root replacement) for IE (n = 37, other group.

**Results:**

The patients in the Ross group included more intravenous drug users and had more prosthetic valve endocarditis and annular abscess formation compared with the other group. Although cardiopulmonary bypass time and aortic cross-clamp time were significantly longer (*P* < .001 and *P* = .003, respectively) and the blood requirements were significantly higher (*P* = .001) in the Ross group, most postoperative short-term outcomes were equivalent between the 2 groups. During follow-up, 1 patient (4%) required reoperation in the Ross group, whereas 6 patients (16%) required reoperation in the other group. Freedom from composite events was significantly better in the Ross group (*P* = .04). Multivariable analysis found the Ross procedure, compared with other procedures, was a significant independent protective factor for composite end points (*P* = .03).

**Conclusions:**

For IE, despite surgical complexity, the Ross procedure yielded short-term outcomes similar to other procedures. In the midterm, the Ross procedure provides a lower reoperation rate, resulting in significantly fewer composite events. The Ross procedure appears to be a better option for patients with IE, but long-term follow-up is necessary.

## Visual Abstract

**Figure:**
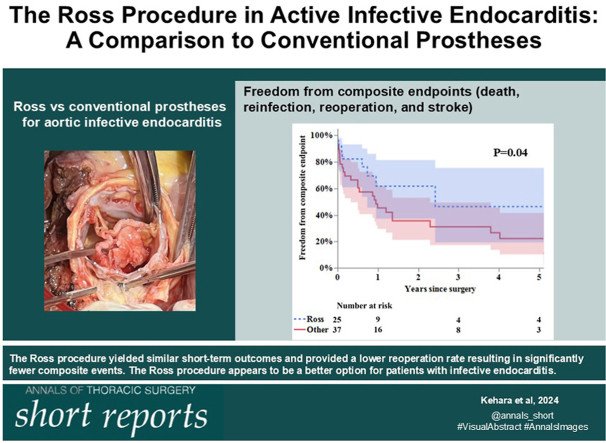

**In Short**

▪ Despite surgical complexity, the Ross procedure yielded similar short-term outcomes.
▪ In the midterm, the Ross procedure provides a lower reoperation rate, resulting in significantly fewer composite events.
▪ The Ross procedure appears to be a better option for patients with infective endocarditis.

Infective endocarditis (IE) is a deadly disease with high mortality and severe complications, despite advances in its management. Surgical treatment is indicated in cases of severe heart failure, uncontrolled infection, such as abscess and fistulas, or to prevent systemic emboli from large or mobile vegetation.^1^^,^^2^ For aortic valve IE, given the reluctance to use foreign materials and their natural biocompatibility, the use of homografts has been suggested to reduce the risk of recurrent infection; however, recent studies have shown no significant benefit.^3^ Guidelines recommend a tailored approach for each situation and do not favor any particular valvular substitute.^1^

The Ross procedure, offering excellent hemodynamic performances and not requiring anticoagulation, has received increasing attention, with data showing restored long-term survival, compared with the general population similar, to that seen in mitral valve repair.^4^^,^^5^ For aortic valve IE, the implantation of an autologous living substitute is an ideal option in patients who are at risk for recurrence, including intravenous drug users (IVDUs).

Although recent studies showed a low operative risk and good long-term results,^6^^,^^7^ no reports to date have compared those outcomes with conventional prostheses specifically in this setting. The purpose of this study was to compare the outcomes of the Ross procedure with those in conventional prostheses in the setting of active aortic valve IE.

## Patients and Methods

Between September 2011 and September 2022, 62 patients underwent aortic valve surgery for active IE at our institution and were included in this study. The diagnosis of active IE was made based on the modified Duke criteria.^8^ The patients were categorized according to the type of implanted aortic valve: autograft (Ross group) and others, including bioprosthetic valves, mechanical valves, and homografts (other group). This study was approved on July 11, 2022, by the Temple University Institutional Review Board (Protocol number: 29747). Patient consent requirements were waived due to minimal risk of the study. Surgical technique and statistical methods for data analysis are described in the Supplemental Material.

## Results

### Patient Characteristics

The baseline characteristics are summarized in Table 1. Whereas 25 patients underwent the Ross procedure (Ross group), 37 patients underwent other procedures (other group). The median age of the Ross group and the other group was 50 years (interquartile range, 38-54 years) and 51 years (interquartile range, 38-61 years), respectively. Fourteen patients (56%) were IVDUs in the Ross group and 17 patients (46%) in the other group. The patients in the Ross group had more prosthetic valve endocarditis compared with the other group, but the difference was not significant (32% vs 14%, *P* = .11). *Streptococcus* spp and *Enterococcus* were the most common causative pathogens in the Ross group, and *Streptococcus* spp was the leading cause in the other group, followed by methicillin-susceptible *Staphylococcus aureus* (*P* = .04).

**Table 1:** Baseline Characteristics

Variable	Ross (n = 25)	Other (n = 37)	*P* Value
Age, y	50 (38-54)	51 (38-61)	.28
Male sex	21 (84%)	27 (73%)	.37
Body mass index, kg/m^2^	23.9 (21.2-29.4)	23.9 (20.4-28.5)	.98
Race			.66
White	10 (40)	14 (38)	
Black	10 (40)	13 (35)	
Asian	0 (0)	1 (3)	
Other	3 (12)	8 (22)	
Unknown	2 (8)	1 (3)	
Hemodialysis	2 (8)	8 (22)	.18
Smoking history	23 (92)	28 (76)	.17
Intravenous drug use	14 (56)	17 (46)	.44
Recent embolic stroke	8 (32)	7 (19)	.24
NYHA Functional Class III or IV	13 (52)	27 (73)	.09
Cardiogenic shock	1 (4)	4 (11)	.64
Emergency surgery	2 (8)	3 (8)	>.99
Prosthetic valve endocarditis	8 (32)	5 (14)	.11
Left ventricular ejection fraction	0.55 (0.50-0.62)	0.58 (0.52-0.63)	.41
Grade >2 aortic insufficiency	18 (72)	31 (84)	.26
Bacteriology			.04
*Streptococcus* spp	6 (24)	13 (35)	
*Staphylococcus aureus*			
Methicillin-susceptible	1 (4)	9 (24)	
Methicillin-resistant	3 (12)	3 (8)	
*Enterococcus*	6 (24)	3 (8)	
Other	3 (12)	7 (19)	
Negative culture	6 (24)	2 (5)	
STS-PROM score, %	5.08 (2.89-7.22)	6.54 (3.09-11.88)	.20

Data are presented as n (%) or median (interquartile range).

NYHA, New York Heart Association; STS-PROM, Society of Thoracic Surgeons predicted risk of mortality.

### Operative Findings

Intraoperative findings are summarized in Table 2. The patients in the Ross group had a notably higher incidence of annular abscess than the other group (56% vs 32%, *P* = .07). In the other group, aortic valve replacement was performed in 31 patients (84%), comprising 27 biological and 4 mechanical valves, and aortic root replacement was performed in 6 patients (16%), consisting of 1 biological, 1 mechanical, and 4 homografts. Cardiopulmonary bypass (CPB) time and aortic cross-clamp time were significantly longer and the blood requirements were significantly higher in the Ross group than those in the other group.

**Table 2:** Operative Findings

Variable	Ross (n = 25)	Other (n = 37)	*P* Value
Bicuspid aortic valve	3 (12)	5 (14)	>.99
Annular abscess	14 (56)	12 (32)	.07
Aortic procedure	…		…
Aortic valve replacement		31 (84)	
Biological		27 (73)	
Mechanical		4 (11)	
Aortic root replacement		6 (16)	
Biological		1 (3)	
Mechanical		1 (3)	
Homograft		4 (11)	
Concomitant surgery			
Mitral valve replacement/repair	4 (16)	11 (30)	.25
Tricuspid valve replacement/repair	3 (12)	8 (22)	.50
Coronary artery bypass grafting	1 (4)	2 (5)	>.99
Cardiopulmonary bypass time, min	208 (176-272)	139 (91-191)	<.001
Aortic cross-clamp time, min	128 (111-157)	90 (72-137)	.003
Transfusion,^a^ total units	12 (5-19)	4 (3-11)	.001

Data are presented as n (%) or median (interquartile range).

a Includes red blood cells, fresh frozen plasma, platelets, and/or cryoprecipitate.

### Early Outcomes

There were 3 hospital deaths (12%) in the Ross group and 5 (14%) in the other group. Causes of death included cardiac arrest (n = 1), bleeding (n = 1), and septic shock (n = 1) in the Ross group and bleeding (n = 2), septic shock (n = 2), and low output syndrome (n = 1) in the other group. Most posttransplant short-term outcomes were equivalent between the 2 groups (Supplemental Table 1).

### Midterm Outcomes

**Supplementary Figure 1:**
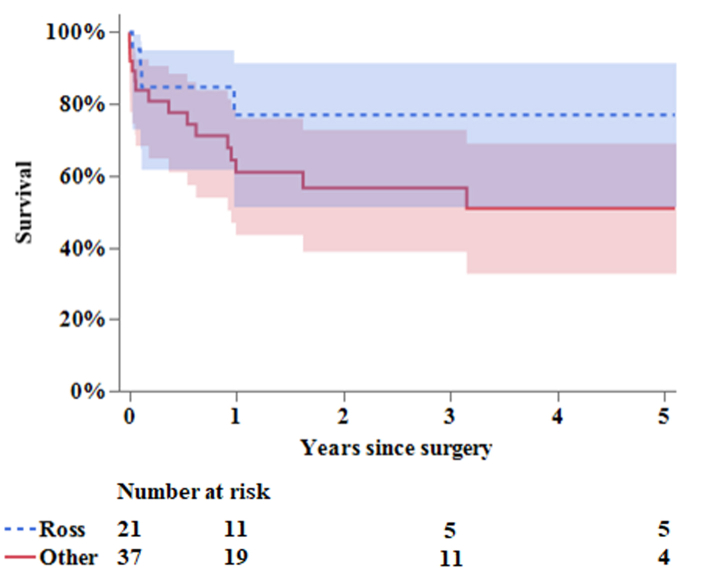


During a mean follow-up of 2.5 ± 2.7 years, 5 patients (20%) died in the Ross group, and 16 (43%) in the other group (Table 3). Survival was worse in the other group compared with the Ross group, but the difference was not significant (*P* = .19) (Supplemental Figure 1).

**Table 3:** Midterm Outcomes

Variable	Ross (n = 25)	Other (n = 37)	*P* Value
Overall death	5 (20)	16 (43)	.10
Valve-related events			
Reinfection	5 (20)	11 (30)	.56
Reoperation	1 (4)	6 (16)	.23
Stroke	1 (4)	2 (5)	>.99
Composite of death and valve-related events	9 (36)	23 (62)	.04

Data are presented as n (%).

Reoperations were required in 6 patients (16%) in the other group and in 1 patient (4%) in the Ross group (Supplemental Table 2). The patient who required the reoperation in the Ross group had prosthetic valve endocarditis in the pulmonary homograft 0.8 years after the initial operation and underwent redo pulmonary valve replacement by pulmonary homograft. Of note, 2 hospital deaths were related to reoperations in the other group. The freedom from reoperation at 1 and 3 years was 90.9% ± 8.7% in the Ross group and 86.6% ± 7.3% in the other group, respectively (Supplemental Figure 2).

Freedom from the composite end point of death, reinfection, reoperation, and stroke at 1 and 3 years was 61.8% ± 12.1% and 46.3% ± 16.2% in the Ross group, respectively, and was 45.3% ± 8.5% and 31.1% ± 8.4% in other group, respectively (Figure). Freedom from the composite end point was significantly better in the Ross group than in the other group (*P* = .04).

**Figure:**
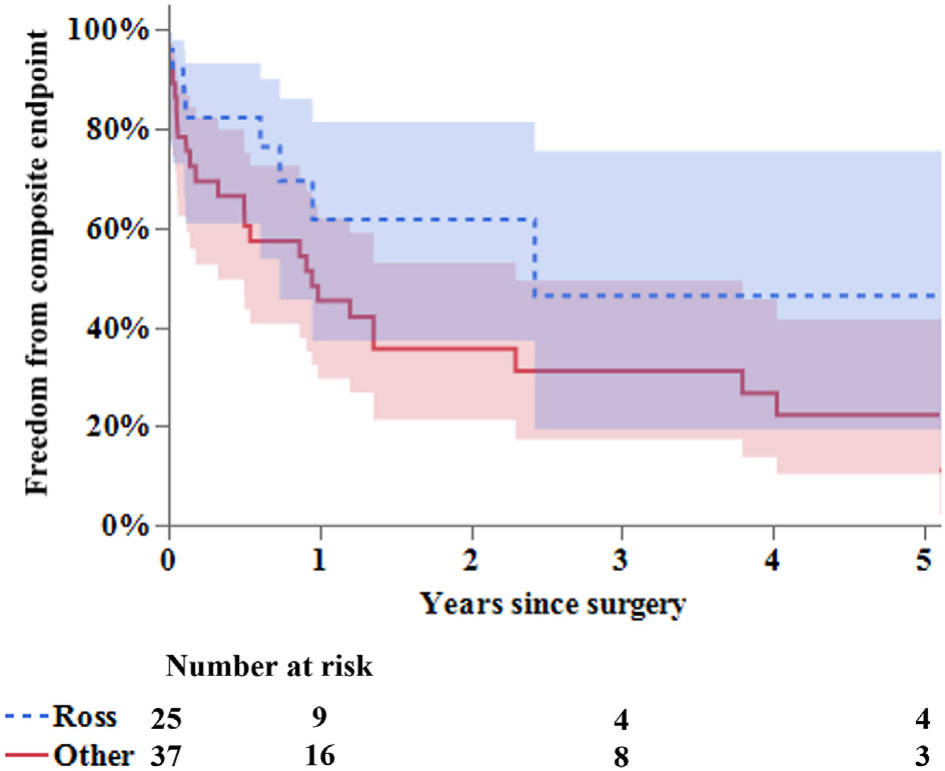
Freedom from the composite end point was significantly better in the Ross group than in the other group (*P* = .04). The shaded areas indicate the 95% CI.

### Factors Associated With Composite End Point

Multivariable analysis showed IVDU, causative pathogen of methicillin-resistant *Staphylococcus aureus*, and the Ross procedure compared with other procedures were significant independent factors for composite end points (Supplemental Table 3).

## Comment

Several recent studies have showed low operative risk and good long-term outcomes regarding survival and recurrent endocarditis with the Ross procedure for aortic valve IE. Loobuyck and colleagues^6^ reported an estimated overall survival and freedom from recurrent endocarditis or reoperation of 84.2% ± 6.6% and 89.4% ± 5.9% at 10 years, respectively, with a median follow-up of 12 years. Chauvette and colleagues^7^ reported that actuarial survival and cumulative incidence of IE recurrence were 88% ± 8% and 13% ± 8% at 8 years, respectively. Our comparison of those outcomes between the Ross procedure and conventional prostheses in this report demonstrates the superiority of the Ross procedure for IE compared with conventional prostheses.

One of the shortcomings of the Ross procedure is its surgical complexity. Reece and colleagues^9^ conducted an analysis using The Society of Thoracic Surgeons Adult Cardiac Surgery Database and reported the Ross procedure was associated with significantly higher operative mortality and perioperative morbidity. In the current study, the CPB time and aortic cross-clamp time were significantly longer, and the amount of blood transfusion was significantly higher in the Ross group than those in the other group, reflecting the surgical complexity; however, there were no significant differences between the 2 groups in early mortality and complications. We speculate that our relatively short aortic cross-clamp time of 133 minutes might have contributed to these results. Additionally, harvesting the pulmonary autograft with the beating heart allowed for secure hemostasis of the right ventricular outflow tract, which also mitigated postoperative bleeding complications.

Another main issue with the Ross procedure is autograft dilatation, and various stabilization techniques have been advocated.^10^ In terms of only preventing autograft dilatation, encasing the autograft in a Dacron graft (DuPont) seems to be the most reproducible approach; however, using prosthetic material is hesitated in IE because the Ross procedure itself has the advantage of avoiding prosthetic material. We do not use any prosthetic material, and long-term follow-up is warranted to determine the pros and cons of using prosthetic material in patients with IE.

### Study Limitations

The present study has several limitations. First, the sample size was small, and not all of the patients were monitored in follow-up. The follow-up was short, however, and obtaining long-term follow-up data may be challenging given the high prevalence of IVDU. Second, the other group was heterogenous in surgical procedure and prosthetic valve used. Including only 1 type of surgery and prosthetic valve as a control group would be better, which was aborted due to the small number of each case.

### Conclusions

Despite surgical complexity, the Ross procedure yielded similar short-term outcomes and significantly fewer composite events compared with conventional prostheses in the setting of IE. After adjusting other factors, the Ross procedure was a significant independent factor for composite end points, along with IVDU and the causative pathogen of methicillin-resistant *Staphylococcus aureus*. The Ross procedure appears to be a better option for IE compared with conventional prostheses in the midterm, but further follow-up is necessary.

## Figures and Tables

**Supplementary Figure 2:**