# Supplementary material for: The Ross Procedure in Active Infective Endocarditis: A Comparison With Conventional Prostheses
Source: Ann Thorac Surg Short Rep. 2024 Sep 1;3(1):47–51. doi: 10.1016/j.atssr.2024.08.011 (PMC11910771; doi:10.1016/j.atssr.2024.08.011)
Supplement: Supplementary Material [file mmc1.docx]

**Supplementary materials**

**The Ross Procedure in Active Infective Endocarditis: A comparison to conventional prostheses**

Hiromu Kehara, Mohammed Kashem, Huaqing Zhao, Sebastian A Iturra, Suyog A Mokashi, Ravishankar Raman, Roh Yanagida, Kewal Krishan, Norihisa Shigemura, Yoshiya Toyoda

**Corresponding Author:**

Yoshiya Toyoda, MD, PhD

Division of Cardiovascular Surgery, Lewis Katz School of Medicine at Temple University

Email: Yoshiya.Toyoda@tuhs.temple.edu

PATIENTS AND METHODS

Table S1

Table S2

Table S3

Figure legends

**PATIENTS AND METHODS**

**Surgical technique**

For the Ross procedure, cardiopulmonary bypass was established with ascending aorta and bicaval cannulation. Pulmonary autograft was harvested under beating heart before aortic cross clamp to minimize myocardial ischemic time. Myocardial protection was achieved using cold crystalloid cardioplegia. After aortic valve was inspected, complete debridement of all infected tissues was performed. When present, abscesses were drained and excluded through direct implantation of the autograft within the left ventricular outflow tract. An autograft was implanted using the full root technique in 23 patients (92%) by single interrupted sutures with an autologous pericardial strip. Coronary buttons were re-implanted in the usual fashion. The distal end of the autograft was directly anastomosed to the native ascending aorta. No autografts were encased in a Dacron graft. The right ventricular outflow tract was reconstructed using a cryopreserved pulmonary homograft (CryoValve SG, CryoLife Inc., Kennesaw, GA, USA). The size of the pulmonary homografts was determined to be 3 or 6 mm larger than the measured autograft size.

**Statistical analysis**

Categorical variables are shown as counts (percentages). Continuous variables are shown as the median (interquartile range). Mann-Whitney U test was used for the comparison of continuous variables, and Pearson’s χ2 test or Fisher’s exact test was used for the comparison of categorical variables between the two groups. Overall survival, freedom from reoperation, and freedom from composite endpoint were estimated by the Kaplan-Meier method and compared using the log-rank test. Survival probability with 95% confidence intervals is shown. The composite endpoint was defined as death, reinfection, reoperation, stroke, and bleeding event. The risk factors of composite endpoint were assessed using univariate and multivariable Cox proportional hazards regression models. Variables with P value <0.10 in univariate analysis were incorporated into the multivariable Cox proportional hazards regression models. A P value of less than 0.05 was considered significant. All statistical analyses were conducted with JMP Pro 15 software and SAS 9.4 (SAS Institute, Cary, NC, USA).

| Table S1. Early outcomes | | | |  |
| --- | --- | --- | --- | --- |
| **Variable^a^** | **Ross (n=25)** | **Other (n=37)** | **P value** | |
| Early complication |  |  |  | |
| Re-exploration for bleeding | 1 (4%) | 2 (5%) | >.99 | |
| Prolonged mechanical ventilation (>24 h) | 10 (40%) | 16 (43%) | 0.80 | |
| Re-intubation/tracheostomy | 4 (16%) | 2 (5%) | 0.21 | |
| Stroke | 0 (0%) | 2 (5%) | 0.51 | |
| Permanent pacemaker implantation | 1 (4%) | 6 (16%) | 0.23 | |
| Newly hemodialysis | 1 (4%) | 2 (5%) | >.99 | |
| ICU stay (day) | 4 (3-8) | 4 (2-10) | 0.69 | |
| Hospital stay (day) | 8 (6-17) | 10 (7-24) | 0.34 | |
| Hospital mortality | 3 (12%) | 5 (14%) | >.99 | |
| ^a^ Data are presented as n (%) or median (interquartile range).  ICU, intensive care unit. | | | |  |

| Table S2. Summary of reoperations | | | | | | | | | | | |
| --- | --- | --- | --- | --- | --- | --- | --- | --- | --- | --- | --- |
| **Group** | **Age (years)** | **Sex** | **BMI** | **Causative pathogen** | **IVDU** | **Annular abscess** | **Initial aortic valve procedure** | **Interval (years)** | **Reoperation indication** | **Reoperation Procedure** | **Reoperation mortality** |
| O | 39 | M | 27.2 | *Streptococcus species* | y | n | Aortic root replacement | 5.2 | PVE | Re-AVR | No |
| O | 28 | M | 28.4 | MSSA | y | y | Aortic root replacement | 0.5 | Pseudoaneurysm of the ascending aorta | Ascending aorta replacement | No |
| O | 52 | M | 68.0 | *Streptococcus species* | n | n | AVR | 7.6 | PVE, aortic root abscess | Ross | No |
| O | 62 | M | 28.0 | MRSA | n | n | AVR | 1.0 | PVE, aortic root abscess | Ross | Yes |
| O | 53 | F | 23.3 | MSSA | y | n | AVR | 3.2 | PVE, pseudoaneurysm of the aortic root | Ross | Yes |
| O | 53 | M | 18.8 | *Serratia species* | y | n | AVR | 0.5 | PVE, aortic root abscess | Ross | No |
| R | 27 | F | 23.1 | *Streptococcus species* | y | y | Ross | 0.8 | PVE of the pulmonary homograft | Re-pulmonary valve replacement | No |
| AVR, aortic valve replacement; BMI, body mass index; IVDU, intravenous drug user; MRSA, Methicillin-resistant *Staphylococcus aureus*; MSSA, Methicillin-susceptible *Staphylococcus aureus*; PVE, prosthetic valve endocarditis. | | | | | | | | | | | |

| Table S3. Factors associated with composite endpoint | | |  | |  | |  |  |
| --- | --- | --- | --- | --- | --- | --- | --- | --- |
|  | **Univariate analysis** | | | **Multivariable analysis** | | | | |
|  | **HR (95% CI)** | **P value** | | **HR (95% CI)** | | **P value** | | |
| Age (per year) | 0.98 (0.95-1.01) | 0.14 | |  | |  | | |
| Male (vs female) | 0.43 (0.21-0.88) | 0.02 | | 0.59 (0.28-1.24) | | 0.16 | | |
| Body mass index (per kg/m2) | 0.97 (0.92-1.01) | 0.23 | |  | |  | | |
| Hemodialysis | 1.08 (0.44-2.61) | 0.87 | |  | |  | | |
| Intravenous drug user | 2.11 (1.06-4.23) | 0.03 | | 2.72 (1.29-5.74) | | 0.008 | | |
| Cardiogenic shock | 2.57 (0.89-7.43) | 0.08 | | 2.63 (0.84-8.30) | | 0.10 | | |
| Prosthetic valve endocarditis | 1.02 (0.44-2.38) | 0.96 | |  | |  | | |
| Methicillin-resistant *Staphylococcus aureus* | 4.15 (1.55-11.15) | 0.005 | | 3.89 (1.37-11.03) | | 0.01 | | |
| Multiple valves affected | 1.51 (0.77-2.99) | 0.23 | |  | |  | | |
| Annular abscess | 0.90 (0.45-1.79) | 0.76 | |  | |  | | |
| Ross (compared to other surgery) | 0.44 (0.20-0.98) | 0.05 | | 0.38 (0.17-0.89) | | 0.03 | | |
| CI: confidence interval; HR: hazard ratio | | | | |  | |  |  |

**Figure legends**

**Figure S1.** Overall survival after surgery between two groups with 95% confidence intervals. Survival was worse in the other group compared to the Ross group, but the difference was not significant (P=0.19).

**Figure S2.** Freedom from reoperation at 1 and 3 years was 90.9 ± 8.7% in the Ross group and 86.6 ± 7.3% in the other group, respectively
